# Physical and Mental Effects of Bathing: A Randomized Intervention Study

**DOI:** 10.1155/2018/9521086

**Published:** 2018-06-07

**Authors:** Yasuaki Goto, Shinya Hayasaka, Shigeo Kurihara, Yosikazu Nakamura

**Affiliations:** ^1^ONSEN Medical Science Research Center, Japan Health & Research Institute, 3-1-4 Nihonbashi, Chuo-ku, Tokyo 103-0014, Japan; ^2^Tokyo City University, 8-9-18 Todoroki, Setagaya-ku, Tokyo 158-8586, Japan; ^3^Jichi Medical University, 3311-1 Yakushiji, Shimosa City, Tochigi 329-0498, Japan

## Abstract

Showering is the most common form of bathing worldwide. Whole-body immersion bathing in warm water (~40°C) is common in Japan and exerts sufficient hyperthermic action to induce vasodilatation and increase blood flow, supplying more oxygen and nutrients to the periphery. Cross-sectional studies report better subjective health status with an immersion bathing habit. This randomized controlled trial compared the effects on health of immersion bathing and shower bathing in 38 participants who received 2-week intervention of immersion bathing in warm water (40°C) for 10 min (bathing intervention) followed by 2-week shower bathing without immersion (showering intervention) or vice versa (n = 19 each group). Visual analog scale scores were significantly better for fatigue, stress, pain, and smile and tended to be better for self-reported heath and skin condition after bathing intervention than after showering intervention. The SF-8 Health Survey showed significantly better general health, mental health, role emotional, and social functioning scores. Profile of Mood State scores were lower for stress, tension-anxiety, anger-hostility, and depression-dejection. Immersion bathing, but not shower bathing, exerts hyperthermic action that induces increased blood flow and metabolic waste elimination, which may afford physical refreshment. Immersion bathing should improve both physical and emotional aspects of quality of life.

## 1. Introduction

Lifestyles can vary widely, and several lifestyle factors such as diet, exercise, sleep, alcohol consumption, and smoking are associated with health and survival [1–4]. Another aspect of lifestyle that varies is bathing, several forms of which include bathing in a conventional shower, steam shower, sauna, or bathtub. Bathing in a shower is the most common form of bathing. In Japan, immersion of the whole body in warm water (around 40°C) is a common habit [5, 6]. It is known that the most beneficial effect of so-called immersion bathing is vasodilation induced by hyperthermic action, which results in systemic elevation of the supply of oxygen and nutrients to the periphery and increased elimination of carbon dioxide and metabolic waste materials [7–10].

A survey of bathing practices in Japan revealed that 80% of participants enjoyed bathing (either in a bathtub or shower) and more than 80% reported sensations or feelings of warmth, relaxation, relief from fatigue, and refreshment after immersion bathing [11]. The weekly frequency of bathing varies by season: in summer, shower bathing occurs, on average 4.4 times per week versus immersion bathing at 3.4 times (at 39.4 ± 1.4°C for 9.4 ± 8.8 min); in winter, immersion bathing occurs, on average, 5.0 times per week (at 41.2 ± 1.2°C for 12.9 ± 9.7 min) and shower bathing at 1.6 times [12].

Our previous cross-sectional studies found good subjective health status, sufficient sleep and rest, low levels of stress, and high subjective happiness in individuals who had a habit of bathing in hot water everyday [13, 14]. However, the benefits of certain periods of bathing interventions have not been reported.

This randomized controlled trial assessed the effects of a total of 4 weeks of intervention consisting of 2 weeks of immersion bathing intervention (bathing intervention) and 2 weeks of shower bathing without immersion intervention (showering intervention) in order to compare the physical and mental effects between the two interventions.

## 2. Materials and Methods

### 2.1. Sample Preparation

Subjects were 38 healthy adults (26 women, 12 men; mean age, 45.7 years, SD = 8.4). We recruited the subjects from a Japanese portal site named KARADAKARA, which was an Internet circle where people are interested in health consisting of about 250 thousand people. The Ethics Committee of Japan Health & Research Institute approved the study protocol, and all work was conducted in accordance with the Declaration of Helsinki (1964). Written informed consent was obtained from each subject before commencing the study. This study is registered in the UMIN Clinical Trials Registry (UMIN000006618).

This intervention study was conducted from October 30 to November 26, 2011, to investigate the two bathing methods of immersion bath in hot water (40°C) for 10 min (bathing) and showering without immersion (showering). Subjects were randomized to the groups, and the effects of bathing for 2 weeks and showering for 2 weeks continuously were compared using a cross-over method. No washout was performed.

### 2.2. Assessment Measures

Perceived health is associated with mortality [15], so self-reported health status (health, skin condition, pain, fatigue, stress, and smile in the mirror) was assessed using a 100-mm visual analog scale (VAS; extremely bad 0 [left] to extremely good 100 [right]) after bathing every day during the intervention periods. To assess health and mood states during each intervention period, participants retrospectively completed the Japanese versions of the 8-item Short Form Health Survey (SF-8) and a short form of the Profile of Mood States (POMS), respectively, after each 2-week intervention period. The SF-8 [16] uses single-item scales to assess 8 items: general health, physical functioning, role limitations due to physical health problems, bodily pain, vitality (energy/fatigue), social functioning, mental health, and role limitations due to emotional problems. Physical and Mental Component Summary scores are also calculated. The POMS [17] is used worldwide for the assessment of mood states, measuring the constructs of tension-anxiety, depression-dejection, anger-hostility, fatigue, confusion, and vigor.

### 2.3. Statistical Analysis

The paired t-test was used to compare subjective health status before and after bathing every day and between the 2-week bathing intervention and the 2-week showering intervention periods. The statistical package SPSS 19.0J (IBM, Tokyo, Japan) was used for analysis.

## 3. Results

Subjects of the analysis were 33 of the 38 participants who remained in good health during the 4-week intervention study and completed all measurement items without missing data. Five subjects excluded from this study could not continue this bathing method because of their private matter which is not a health problem.

### 3.1. Self-Reported Health Status

Tables 1 and 2 show the VAS scores for self-reported health status every day before and after the bathing and showering interventions, respectively. Significant improvements were observed for all assessed items after each intervention.

Table 3 shows the results for self-reported health status during each intervention completed retrospectively after each intervention period. VAS scores were higher for self-reported health and skin condition (p < 0.10) and significantly higher for smile (p < 0.05) during bathing intervention than during showering intervention. In addition, fatigue, stress, and pain scores were significantly lower during bathing intervention than during showering intervention (p < 0.05).

### 3.2. Health-Related Quality of Life Assessment

Table 4 shows the individual scores for the 8 items on the SF-8 and the Physical and Mental Component Summary scores indicating health status during each 2-week intervention, as retrospectively self-reported after completing each intervention. General health, social functioning, mental health, and Mental Component Summary scores were significantly higher during bathing intervention than during showering intervention (p < 0.05). There were no differences in role physical, physical functioning, bodily pain, role emotional, or Mental Component Summary scores.

### 3.3. Mood States

Table 5 shows mood states assessed after the 2-week bathing intervention and the 2-week showering intervention, measured using the 6-item POMS. Scores were significantly lower for tension-anxiety, depression-dejection, and anger-hostility during bathing intervention than during showering intervention (p < 0.05). There were no significant differences in fatigue, confusion, or vigor.

## 4. Discussion

This randomized controlled trial comparing the physical and mental effects of 2 weeks of bathing intervention and 2 weeks of showering intervention (without immersion) showed that bathing intervention was more beneficial than showering intervention. The sample size is small, which means this result has limited power. However this is a cross-over study, so that we think the result which we showed is a casual relationship.

Self-assessment of health status before and after intervention every day showed health improvement after both bathing and showering interventions, although the degree of improvement was larger after the former than after the latter. This result suggests that either intervention improves physical and mental condition. Bodily cleanliness and a feeling of refreshment are benefits offered by bathing and showering.

Bathing intervention did, however, show better subjective health status (VAS scores) than showering intervention.

Our previous cross-sectional studies [13, 14] suggested that people who had a habit of bathing in hot water have good subjective health status, sufficient sleep and rest, low levels of stress, and high subjective happiness. The results of this study support that the psychological benefits of bathing are a real causal relationship.

During bathing, several actions unique to bathing will be exerted on the body, including hyperthermic action, hydrostatic pressure, buoyancy, and viscosity of water.

The most important of these is hyperthermic action, which warms the blood in superficial vessels, thereby increasing the deep body temperature through circulation. With an increase in body temperature, heat-sensitive neurons are excited while cold-sensitive neurons are inhibited in the thermoregulatory center of the hypothalamus, causing inhibition of the sympathetic nerves and stimulation of the parasympathetic nerves, leading to vasodilatation and induced perspiration to decrease the body temperature. Heart rate will rise by 40% to 50%, and peripheral pO_2_ will increase while pCO_2_ will decrease, thereby stimulating metabolism and inducing elimination of metabolic waste materials, which in turn refreshes the body [7–10]. In terms of hydrostatic pressure, it induces venous flow, thereby increasing cardiac output and improving metabolism. Also, a habit of immersion bathing in hot water was shown to be associated with strengthened immune function [18, 19].

A bathing study using patients with cardiovascular diseases showed the improvement of hemodynamics by heating effect [20]. On the other hand, for aged generation especially patients for cardiovascular diseases, full body immersion would be unbeneficial because hydrostatic pressure causes venous return load.

This study showed better self-assessment results for fatigue, subjective health, skin condition, and smile as well as a better SF-8 Physical Component Summary score during the intervention with daily immersion bathing, suggesting systemic improvement of metabolism by taking an immersion bath. Furthermore, hyperthermic action is expected to systemically relax the muscles, soften collagen in ligaments and articular capsules, and improve musculoskeletal function. The pain-relieving effect of bathing [21] may explain the reduction in self-rated pain reported by subjects in the present study. Lastly, the downward force of gravity is reduced by buoyancy during bathing, which may in turn lead to the improvements seen in the POMS constructs of tension-anxiety, depression-dejection, and anger-hostility, suggesting positive effects of stress relief, refreshment, and relaxation from immersion bathing.

The limitations of this study are as follows. This trial was conducted in the fall (October-November), and seasonal differences in bathing habit (e.g., autumn versus summer or winter) were not taken into account. Also, the timing of body immersion and the temperature of water in the bathtub were not strictly defined. Interventional studies using tightly controlled conditions will provide insight that is useful for health promotion. In addition, there may be a bias caused by the fact that Japanese likes bathing in general. Even though we think that heating effect of bathing does not differ depending on races, if we do the same intervention study with people in Europe, the result may be changed because the favor regarding bathing may be different between Japanese and people in other countries

## 5. Conclusions

Routine immersion bathing appeared more beneficial to mental and physical health than routine shower bathing without immersion. Further interventional studies that consider seasonal factors and physiological factors in relation to effective bathing temperature and timing are anticipated to show the effect of immersion bathing and clarify the beneficial effects on health.

## Figures and Tables

**Table 1:** Self-reported health status (VAS score) before and after bathing intervention.

	Before			After			Difference	p value	
	Mean		SD	Mean		SD	Mean		
Self-reported health	67.1	±	17.2	77.0	±	13.8	9.9	<0.001	*∗∗*
Skin condition	64.1	±	16.8	76.0	±	13.8	11.9	<0.001	*∗∗*
Fatigue	56.3	±	23.0	40.8	±	24.1	−15.5	<0.001	*∗∗*
Stress	43.9	±	26.3	28.6	±	22.1	−15.3	<0.001	*∗∗*
Pain	20.6	±	26.8	13.9	±	20.3	−6.8	<0.001	*∗∗*
Smile	63.2	±	19.3	75.9	±	16.7	12.6	<0.001	*∗∗*

*∗∗*p<0.01.

**Table 2:** Self-reported health status (VAS score) before and after showering intervention.

	Before			After			Difference	p value	
	Mean		SD	Mean		SD	Mean		
Self-reported health	67.5	±	17.0	73.0	±	16.1	5.5	<0.001	*∗∗*
Skin condition	62.8	±	14.2	69.3	±	13.4	6.5	<0.001	*∗∗*
Fatigue	57.2	±	25.5	48.8	±	25.9	−8.4	<0.001	*∗∗*
Stress	49.0	±	28.2	38.7	±	24.7	−10.2	<0.001	*∗∗*
Pain	24.9	±	28.7	21.9	±	26.8	−3.0	<0.001	*∗∗*
Smile	62.0	±	19.8	70.3	±	20.2	8.3	<0.001	*∗∗*

*∗∗*p<0.01.

**Table 3:** Differences in self-reported health status (VAS score) between bathing and showering interventions.

	Bathing			Showering			Difference	p value	
	Mean		SD	Mean		SD	Mean		
Self-reported health	76.5	±	9.8	71.8	±	14.5	4.6	0.072	
Skin condition	72.5	±	12.0	67.4	±	12.9	5.1	0.053	
Fatigue	42.0	±	20.9	52.2	±	24.6	−10.2	0.028	*∗*
Stress	39.2	±	23.2	50.7	±	25.5	−11.5	0.008	*∗∗*
Pain	16.2	±	20.0	23.5	±	26.2	−7.3	0.040	*∗*
Smile	74.3	±	13.2	68.7	±	13.5	5.6	0.016	*∗*

*∗*p<0.05 and *∗∗*p<0.01.

**Table 4:** Health-related quality of life (SF-8 scores) reported retrospectively for each intervention period.

	Bathing			Showering			Difference	p value	
	Mean		SD	Mean		SD	Mean		
General health	54.5	±	6.2	50.2	±	7.0	4.3	0.010	*∗∗*
Physical functioning	51.2	±	4.2	50.7	±	4.2	0.5	0.576	
Role physical	50.5	±	5.7	50.9	±	5.1	−0.4	0.680	
Bodily pain	50.4	±	8.0	49.4	±	8.8	1.0	0.381	
Vitality	51.2	±	6.6	49.2	±	5.8	2.1	0.110	
Social functioning	50.2	±	7.8	46.4	±	7.3	3.8	0.016	*∗*
Mental health	49.9	±	6.5	47.0	±	5.7	2.9	0.021	*∗*
Role emotional	49.6	±	7.7	48.3	±	4.7	1.3	0.358	
Physical component summary	50.8	±	6.0	50.4	±	6.0	0.4	0.691	
Mental component summary	48.9	±	6.6	45.6	±	5.4	3.3	0.010	*∗∗*

*∗*p<0.05 and *∗∗*p<0.01.

**Table 5:** Retrospectively assessed mood states during each intervention (POMS scores).

	Bathing			Showering			Difference	p value	
	Mean		SD	Mean		SD	Mean		
Tension-Anxiety	43.7	±	6.2	46.5	±	8.1	−2.9	0.010	*∗*
Depression-Dejection	47.5	±	8.7	49.6	±	8.5	−2.1	0.042	*∗*
Anger-Hostility	46.9	±	6.8	50.0	±	9.7	−3.1	0.024	*∗*
Fatigue	46.2	±	7.1	48.1	±	8.6	−1.8	0.124	
Confusion	48.2	±	8.4	49.6	±	8.6	−1.4	0.329	
Vigor	51.6	±	10.8	50.4	±	8.2	1.2	0.495	

*∗* p<0.05.

## Data Availability

The data used to support the findings of this study were provided by Japan Health & Research Institute under license and so cannot be made freely available. Access to these data will be considered by the author upon request, with permission of Yasuaki Goto.
